# Advancing Youth Participation to Inform Equitable Health Policy: Comment on "Between Rhetoric and Reality: Learnings From Youth Participation in the Adolescent and Youth Health Policy in South Africa"

**DOI:** 10.34172/ijhpm.2023.7974

**Published:** 2023-08-08

**Authors:** Janet Njelesani, Jean Hunleth

**Affiliations:** ^1^Department of Occupational Therapy, New York University (NYU), New York City, NY, USA.; ^2^Division of Public Health Sciences, Washington University in St. Louis, St. Louis, MO, USA.

**Keywords:** Youth Participation, Policy Development, Participatory Research, Action Research, Health, South Africa

## Abstract

In their published study, Jacobs and George examine how youth participation was enabled to advance the Adolescent and Youth Health Policy (AYHP) in South Africa. Using an expanded and adapted conceptual framework of youth participation to inform their work, their findings center on the complexities of youth participation including enablers and the challenges experienced in the South African context. Building upon their foundational work, in this commentary we suggest further insights for consideration to advance youth participation to inform equitable health policies, including the inclusion of youth with intersecting identities and critical reflection to further advance the adapted conceptual framework.

## Introduction

 “*It was very clear that we weren’t getting to what I thought were the key issues. So, we then switched gears and said, well, let’s ask young people*” (AYHP Author Government 14). This recognition from a Government representative in the published paper, “Between Rhetoric and Reality: Learnings From Youth Participation in the Adolescent and Youth Health Policy in South Africa,”^1^ illuminates the essential contribution that youth can make in policy development. The participation of youth offers a unique contribution that cannot be obtained through alternative methods such as interviewing adults, because adults, including parents, are inadequate proxies for capturing the perceptions and perspectives of young people. If youth do not participate in informing policy that affects their lives, the developed policies may be ineffective, detrimental, and insensitive to their rights, needs, and experiences.^2^ Youth participation in policy development is crucial for understanding key issues and creating appropriate and impactful policies.^3^ The absence of youth participation in policy development leads to the creation of inappropriate or ineffectual policies, as it overlooks the firsthand experiences, perspectives, and needs of young individuals, resulting in a disconnect between policy decisions and the realities faced by the youth population. In their 2022 paper, Jacobs and George examined youth participation in the Adolescent and Youth Health Policy (AYHP) development process to understand how youth were included in health policy-making in the South African context. Jacobs and George use the term “youth” in their paper when referring to young people between the ages of 10 and 24, and we use the same term in this commentary to maintain definitional congruity. The published work adds an important contribution to the current literature on youth participation to inform health policy with many novel insights highlighted in this commentary. In particular, Jacobs and George’s consideration of perspectives of youth living in a middle-income country is important as these perspectives have received less scholarly attention. The majority of previously published work has centered on adult perspectives and people from high-income countries.^4^

 Another novel idea brought forth in the work is the adaptation of Cahill and Dadvand’s P7 Model. The P7 Model is a framework for conceptualizing and planning youth participation that accounts for socio-cultural contexts.^5^ The original P7 Model of Cahill and Dadvand presents the seven inter-connected domains of *Purpose, Place, Process, Positioning, Protection, Perspective*, and *Power relations*. Jacobs and George expanded the model by adding the two additional domains of *People* and Partnerships, with an accompanying set of new questions. The authors used the adapted conceptual framework to identify the key successes and challenges in policy development that arose during youth participation in the AYHP policy formulation process which may be applicable to other settings. Key findings included that youth participation in the AYHP process was challenged within the fragmented policy landscape and youth participation was supported by academic and government actors with a history of collaborating with youth. These findings add to the evidence-base on the need to support and strengthen capacities of decision-makers and researchers to engage youth in policy, research, and practice.

## Building on the Conceptual Framework

 The addition of People and Partnerships into the P7 Model by Jacobs and George proved useful to highlight multi-sector partnerships and how working across government departments proved challenging and hindered youth involvement, a key finding of the study. The addition of the two new domains also enabled a deeper examination of the roles of each of the policy actors within the policy development context. An important finding included that in systems without organized youth health actors, youth participation was challenged by lack of coordination between youth stakeholders.

 The guiding questions derived from the adapted P7 Model, as seen in Box 2 in the original article, are also valuable contributions to the literature as they can be used to guide work that integrates youths’ perspectives into policy. Jacobs and George used the guiding questions to identify how government departments were working in silos which created a lack of synergy and negated a shared vision for youth participation.We are excited about how these questions could be incorporated into our work that advances youth participation. Efforts aimed at enhancing health policy development participation with adolescents can serve as a model for similar initiatives with younger children, as they highlight the importance of including young individuals in decision-making processes, ensuring their voices are heard, and addressing their unique healthcare needs. We envision the guiding questions being juxtaposed alongside the reflective questions previously developed by us (Figure) to delve even deeper into issues of meaningful inclusion in research and policy development within the context in that young people live. The Reflective Guide was created as a tool for health researchers to enhance the meaningfulness of children’s participation. Considering the shared goal of health policy-makers, implementing the strategies outlined in the Guide would be appropriate to integrate children and youth’s perspectives more effectively into their work. The Guide primarily emphasizes the importance of recognizing and addressing trends and gaps, improving methodological clarity, diversifying methodological approaches, and addressing power structures that impede meaningful participation of children. Although initially developed for children aged 6-18, the Guide’s principles and recommendations are applicable and relevant to a broader age range, including older youth.

**Figure:**
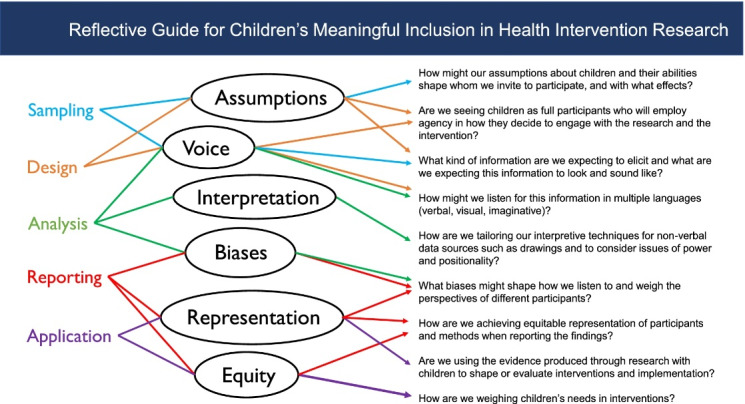
Reflective Guide for Children’s Meaningful Inclusion.

 Cahill and Dadvand’s P7 Model was designed to illuminate how the context and intersecting social and structural determinants, including colonialism, apartheid, poverty, and racial and gender inequality influence youth participation. Using this intersectional lens, one line of inquiry that could have been examined further by Jacobs and George was how youth participation influenced empowerment and how engagement may have reinforced intersecting inequities. Having an enhanced understanding of these issues would be beneficial to know what participatory strategies to build upon and which to avoid to reduce harm when developing future policies with youth.

## Diverse Youth Perspectives

 “*When thinking about policy-making processes we need to ask how participation was gained and how different constituencies of adolescents and youth were considered*” (AYHP Advisory Panel Member 11).Participants recognized that the policy development process did not involve a diverse array of youth. The authors also recognized this concern in their findings and noted how diverse youth engagement was a key challenge in the policy development process. Importantly, the study’s findings show how the policy development process did not include youth with intersecting identities (ie, age, disability, sexuality, and geographical settings) due to broader contextual challenges including resource limitations. These findings make evident the need to advocate for more resources and time to better include youth in policy development so as not to tokenize youth participation. To address these challenges, recommendations in the paper point to the need for health policy decision-makers to systematically include diverse youth in policy-making. They recognize this action is “both an ambitious goal and a vexing challenge to implement in reality” (p. 9). We agree that a lot of work needs to be done, and from our previous work collaborating with youth to facilitate youth participation, we suggest strategies below under future directions to engage youth in policy, research, and practice in meaningful ways.

 When thinking about who is included, we would have liked to better understood about how Jacobs and George accounted for diverse youth participation in their study. Despite the current study focusing only on youth who were involved in the AYHP process, we suggest that opportunities to include more youth in the study may have been missed. The identities (eg, gender, age, race, ethnicity, sexuality, and disability) of youth participants were not shared nor was a reflection on how those identities intersected with social and structural determinants in South Africa. Furthermore, there was no indication in the methods section of how methods were potentially adapted to meet the needs and strengths of youth, in particular youth requiring accommodations to enable participation. We wonder if the same findings about challenges to youth involvement affected the research and, if so, what further research on the AYHP, inclusive of more young people, might reveal about the findings. This limitation of the study is noted by the authors, when they write, the study “does not include perspectives of representative and diverse youth and structures in the general population”; however, no suggestions were provided by them for how to mitigate this limitation in the future.

## Future Directions

 Jacobs and George show us that a lot more work needs to be done to include rather than tokenize youth in policy development. Jacobs and George highlighted particular challenges of engaging with youth with intersecting identities. Here we unpack future considerations to inform equitable policies grounded in the perspectives of youth, focusing on our areas of study with youth with disabilities and young children.

 Future work that focuses on studying youth representation in the policy development process could be more inclusive by gathering a diverse range of youth perspectives.By not including the perspectives of a diverse range of youth in policy development, decision-makers are inadvertently contributing to societal exclusion of youth from historically under-represented groups. Youth with disabilities have a right to access health services and may need greater services as a result of living with a health condition or impairment in a low-resourced setting.^6,7^ They should be included in health policy decisions that impact their daily lives and right to health. One solution is drawing from the field of disability studies to enhance the inclusion of youth with disabilities’ perspectives using culturally attuned and inclusive methods. Best practice includes adapting and including an array of multi-method inclusive, accessible, adaptable, and non-ableist tools (eg, photo elicitation, cartoon captioning, vignettes, sentence starters, drawing) to enable different ways of expression.^8^ It is important to include the perspectives of a diverse range of youth, including youth with disabilities, when developing policies applicable to all children (eg, school and housing). When the rights of youth with disabilities are siloed into disability policy, decision makers are not recognizing them as holistic individuals with diverse needs that cross-cut all sectors.

 Including diverse perspectives of youth also includes understanding how to include younger children (ie, less than 10 years of age) using approaches that are tailored to younger children’s needs and social position. The perspectives of younger children have not always been included in decision making processes despite the existing evidence about the unique perspective that their inclusion provides.^4^ Literature from childhood studies offers a breadth of approaches that attend to power structures for engaging younger children, from ethnography to arts-based approaches.^9,10^ Meaningful participation can take many forms including such as the use of reflective guides to attend to representation, voice, interpretation, biases, representation, and equity.^4^ No matter the strategies used, the aim is always to listen to younger children without tokenizing them or treating them as passive subjects to study.

 Further work could reflect on if another schematic of the P7 Model would better capture the intended connections across domains versus the current centering of Purpose. We wonder if the domain of Power would be most important to inform the design of youth participatory processes in policy development. With Purpose centered, the current study’s focus on adults’ perspectives in the work is apparent. The study primarily engaged with people in positions of power and authority, with less youth interviewed (a total of 3 out of 30 participants) and fewer quoted.However,adults are not good proxies for young people’s perceptions. The work could also be strengthened by acknowledging how the work benefitted adult researchers and being reflexive about how adult responses were prioritized.^9^ Further, in the reflexivity section, the unique power differential between the researchers and youth participants was recognized to a lesser degree. Greater recognition of power differentials would provide an opportunity to critically examine what was said by youth but never followed up on, what issues and by whom got put in the foreground of policy discussions, what and whose ideas were marginalized, and for whom did the policy formation process benefit most.

## Conclusion

 In this commentary, we critically reflect on the use of the P7 Model adapted by Jacobs and George to examine youth participation in the development of the AYHP in South Africa. We commend their attention to and support of youth participation in health policy development in a place and space where youth have not historically been involved. We suggest strategies to build off the work of Jacobs and George to ensure future health policy development centers youth perspectives and is more inclusive of a diversity of youth.

## Ethical issues

 Not applicable.

## Competing interests

 Authors declare that they have no competing interests.

## Funding

 This work is supported in part by an American Occupational Therapy Foundation (AOTF) Implementation Research Grant (**Error! Hyperlink reference not valid.**) received by JN and JH. JH’s work on this article was also funded, in part, by the National Cancer Institute, grant number U01CA27503. The funders had no role in the decision to publish or in the preparation of the manuscript.

## References

[1] Jacobs T, George A (2022). Between rhetoric and reality: learnings from youth participation in the Adolescent and Youth Health Policy in South Africa. Int J Health Policy Manag.

[2] Njelesani J, Hunleth J (2020). Youth participatory research evidence to inform health policy: a systematic review protocol. BMJ Open.

[3] Spray J (2018). The value of anthropology in child health policy. Anthropol Action.

[4] Hunleth JM, Spray JS, Meehan C, Lang CW, Njelesani J (2022). What is the state of children’s participation in qualitative research on health interventions?: a scoping study. BMC Pediatr.

[5] Cahill H, Dadvand B (2018). Re-conceptualising youth participation: a framework to inform action. Child Youth Serv Rev.

[6] Njelesani J, Cameron D, Gibson BE, Nixon S, Polatajko H (2014). A critical occupational approach: offering insights on the sport-for-development playing field. Sport Soc.

[7] Njelesani J, Sedgwick A, Davis JA, Polatajko HJ (2011). The influence of context: a naturalistic study of Ugandan children’s doings in outdoor spaces. OccupTher Int.

[8] Njelesani J, Mlambo V, Denekew T, Hunleth J (2022). Inclusion of children with disabilities in qualitative health research: a scoping review. PLoS One.

[9] Hunleth J (2011). Beyond on or with: questioning power dynamics and knowledge production in ‘child-oriented’ research methodology. Childhood.

[10] Clark CD. In a Younger Voice: Doing Child-Centered Qualitative Research. New York: Oxford University Press;.

